# Supplementary material for: DNA methylation memory of pancreatic acinar-ductal metaplasia transition state altering Kras-downstream PI3K and Rho GTPase signaling in the absence of Kras mutation
Source: Genome Med. 2025 Mar 28;17:32. doi: 10.1186/s13073-025-01452-6 (PMC11951614; doi:10.1186/s13073-025-01452-6)
Supplement: Supplementary file 1 — Supplementary Material 1 [file 13073_2025_1452_MOESM1_ESM.zip › ADM_Additional_Files_Compiled.pdf]

DNA methylation memory of pancreatic acinar-ductal metaplasia transition state altering Kras-downstream PI3K and Rho GTPase signaling in the absence of Kras mutation

Emily K.W. Lo[1,2,3,4], Adrian Idrizi[2,4], Rakel Tryggvadottir[2,4], Weiqiang Zhou[5], Wenpin Hou[5], Hongkai Ji[5], Patrick Cahan[1,3,*], Andrew P. Feinberg[1,2,4*]

Additional Files

Supplementary Tables S1-8

Supplementary Figures 1-10

Supplementary Tables

Table S1. (Additional File 1)
Animal cohorts.

Table S2. (Additional File 2)
qPCR primer sequences.

Table S3. (Additional File 3)
MERFISH 500-gene target panel comprising a mixture of canonical marker genes, differentially methylated genes identified via WGBS, differentially expressed genes identified via 10X Visium ST, and TFs identified via differential motif enrichment analysis.

Table S4. (Additional File 4)
Publicly available single-cell mouse pancreas studies mined from NCBI Gene Expression Omnibus (GEO) used for deconvolution of spatial transcriptomics data.

Table S5. (Additional File 6)
Summary of pancreatic samples and omics QC metrics.

Table S6. (Additional File 7)
Genomic variants for all AK experiment mice as called by freebayes and maftools.

Table S7. (Additional File 11)
Genes overlapping differentially methylated regions (DMRs) for:

- AK D2 ADM (n = 2 mice) vs normal acini (n = 4 mice)
- AK D4 ADM (n = 2 mice) vs normal acini (n = 4 mice)
- AK D7 (recovered, n = 2 mice) vs normal acini (n = 4 mice), all
- AK D7 (recovered, n = 2 mice) vs normal acini (n = 4 mice), where the D7 methylation level was more extreme than that of D2 and D4
- Caerulein D2 ADM (n = 2 mice) vs normal acini (n = 4 mice)
- Caerulein D4 ADM (n = 2 mice) vs normal acini (n = 4 mice)
- Caerulein D7 (recovered, n = 2 mice) vs normal acini (n = 4 mice)

Positive areaStat: hypermethylated in group 1.

Table S8. (Additional File 13)
Homer motif analysis results for AK D4 and Caerulein D2 comparisons.

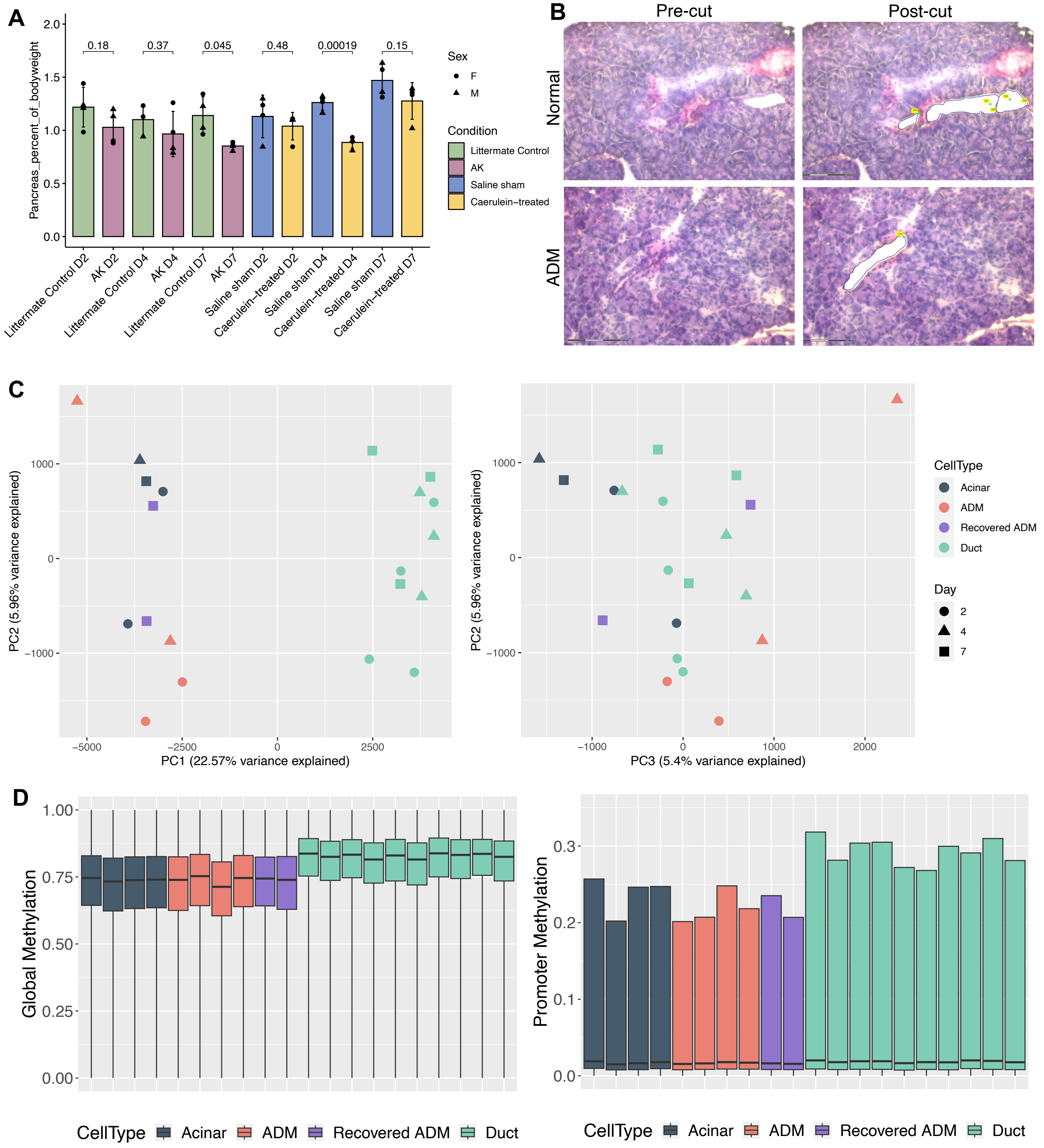
A
B
C
D
Pancreas_percent_of_bodyweight
0.0
0.5
1.0
1.5
2.0
0.18
0.37
0.045
0.48
0.00019
0.15
Littermate Control D2
AK D2
Littermate Control D4
AK D4
Littermate Control D7
AK D7
Saline sham D2
Caerulein−treated D2
Saline sham D4
Caerulein−treated D4
Saline sham D7
Caerulein−treated D7
Sex
F
M
Condition
Littermate Control
AK
Saline sham
Caerulein−treated
Pre-cut
Post-cut
Normal
ADM
PC2 (5.96% variance explained)
PC1 (22.57% variance explained)
PC3 (5.4% variance explained)
1000
0
−1000
−5000
−2500
0
2500
−1000
0
1000
2000
CellType
Acinar
ADM
Recovered ADM
Duct
Day
2
4
7
Global Methylation
0.00
0.25
0.50
0.75
1.00
Promoter Methylation
0.0
0.1
0.2
0.3
CellType
Acinar
ADM
Recovered ADM
Duct
CellType
Acinar
ADM
Recovered ADM
Duct

Figure S1. (Additional File 5). Laser-capture microdissection (LCM) and whole-genome bisulfite sequencing (WGBS) of exocrine cell types in mouse models of ADM.

A. Representative examples of pre- and post-LCM cuts for ducts isolated from normal and ADM H&E-stained tissue.

B. Principal component analysis (PCA) of genome-wide CpG methylation for all exocrine samples in AK model of ADM.

C. Boxplots of genome-wide (left) and promoter-specific (right) CpG methylation values for all exocrine samples in AK model of ADM. Center line, median; box limits, upper and lower quartiles; whiskers, 1.5x interquartile range.

Normal acinar, n = 4 mice; day 2 ADM, n = 2 mice; day 4 ADM, n = 2 mice; day 7 ADM, n = 2 mice; duct, n = 10 mice.

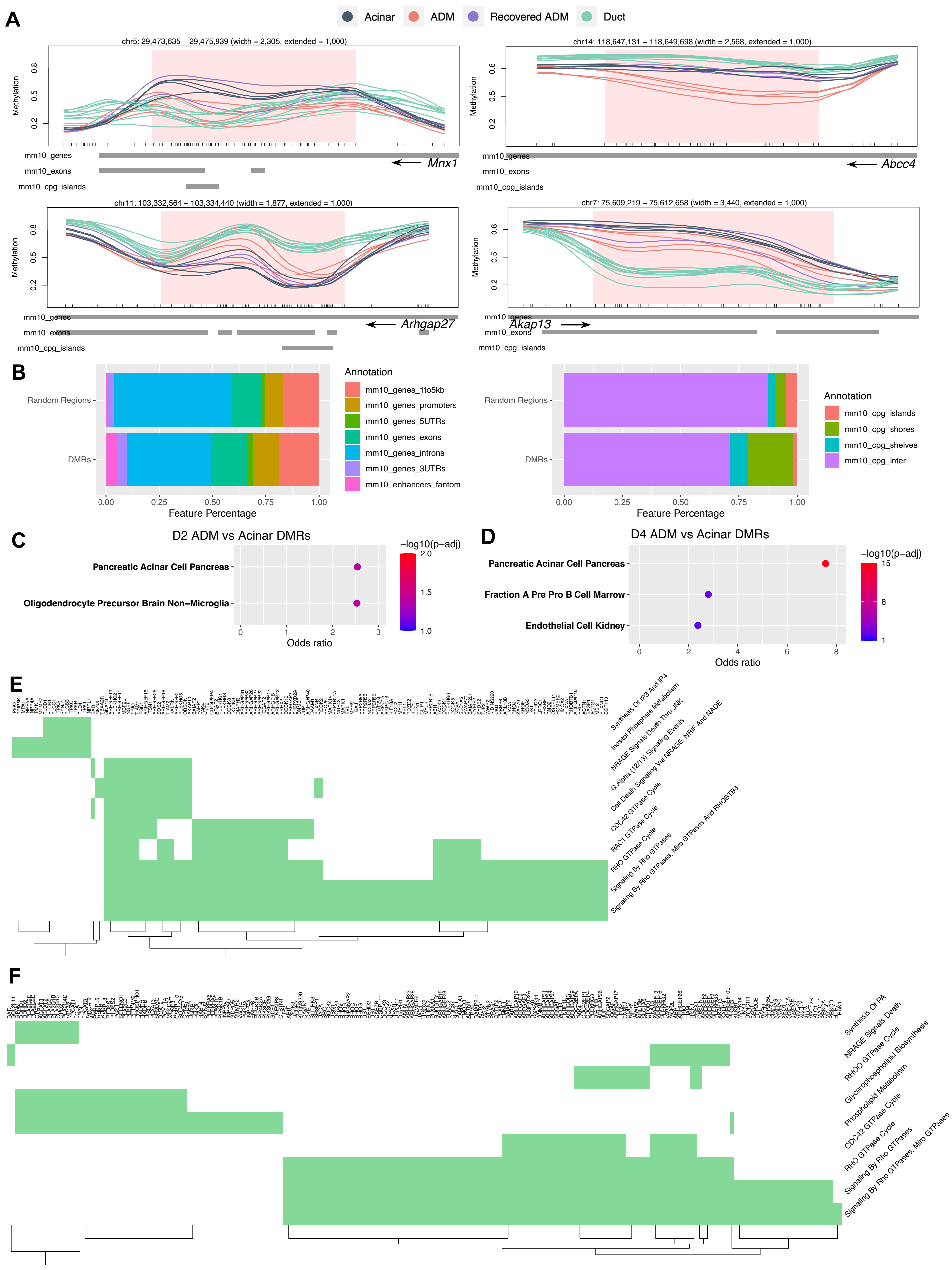
A
Acinar
ADM
Recovered ADM
Duct
chr5: 29,473,635 – 29,475,939 (width = 2,305, extended = 1,000)
chr14: 118,647,131 – 118,649,698 (width = 2,568, extended = 1,000)
chr11: 103,332,564 – 103,334,440 (width = 1,877, extended = 1,000)
chr7: 75,609,219 – 75,612,658 (width = 3,440, extended = 1,000)
Methylation
mm10_genes
mm10_exons
mm10_cpg_islands
Mnx1
Abcc4
Arhgap27
Akap13
B
Random Regions
DMRs
Feature Percentage
Annotation
mm10_genes_1to5kb
mm10_genes_promoters
mm10_genes_5UTRs
mm10_genes_exons
mm10_genes_introns
mm10_genes_3UTRs
mm10_enhancers_fantom
mm10_cpg_islands
mm10_cpg_shores
mm10_cpg_shelves
mm10_cpg_inter
C
D2 ADM vs Acinar DMRs
Pancreatic Acinar Cell Pancreas
Oligodendrocyte Precursor Brain Non−Microglia
Odds ratio
−log10(p−adj)
D
D4 ADM vs Acinar DMRs
Pancreatic Acinar Cell Pancreas
Fraction A Pre Pro B Cell Marrow
Endothelial Cell Kidney
Odds ratio
−log10(p−adj)
E
Synthesis Of IP3 And IP4
Inositol Phosphate Metabolism
NRAGE Signals Death Thru JNK
G Alpha (12/13) Signaling Events
Cell Death Signaling Via NRAGE, NRIF And NADE
CDC42 GTPase Cycle
RAC1 GTPase Cycle
RHO GTPase Cycle
Signaling By Rho GTPases
Signaling By Rho GTPases, Miro GTPases And RHOBTB3
F
Synthesis Of PA
NRAGE Signals Death
RHOQ GTPase Cycle
Glycerophospholipid Biosynthesis
Phospholipid Metabolism
CDC42 GTPase Cycle
RHO GTPase Cycle
Signaling By Rho GTPases
Signaling By Rho GTPases, Miro GTPases

Figure S2. (Additional File 8). ADM vs acinar DMR profiles in AK ADM model.

A. Genomic line plots of methylation level at representative DMRs comparing ADM lesions (D2 and D4) to normal acini. Tick marks on horizontal axes represent individual CpG sites. Normal acinar, n = 4 mice; day 2 ADM, n = 2 mice; day 4 ADM, n = 2 mice; day 7 ADM, n = 2 mice; duct, n = 10 mice.

B. Stacked barplot of genomic region distribution of AK ADM vs acinar control DMRs as compared to randomly selected genomic regions of the same sizes.

C. Fisher's GSEA of genes overlapping AK model D2 ADM vs acinar DMRs using Tabula Muris gene sets.

D. Fisher's GSEA of genes overlapping AK model D4 ADM vs acinar DMRs using Tabula Muris gene sets.

E. Genes (rows) overlapping AK D2 ADM vs normal acinar DMRs that contribute to each significantly enriched gene set (columns) as in Fig. 2C.

F. Genes (columns) overlapping AK D4 ADM vs normal acinar DMRs that contribute to each significantly enriched gene set (rows) as in Fig. 2D.

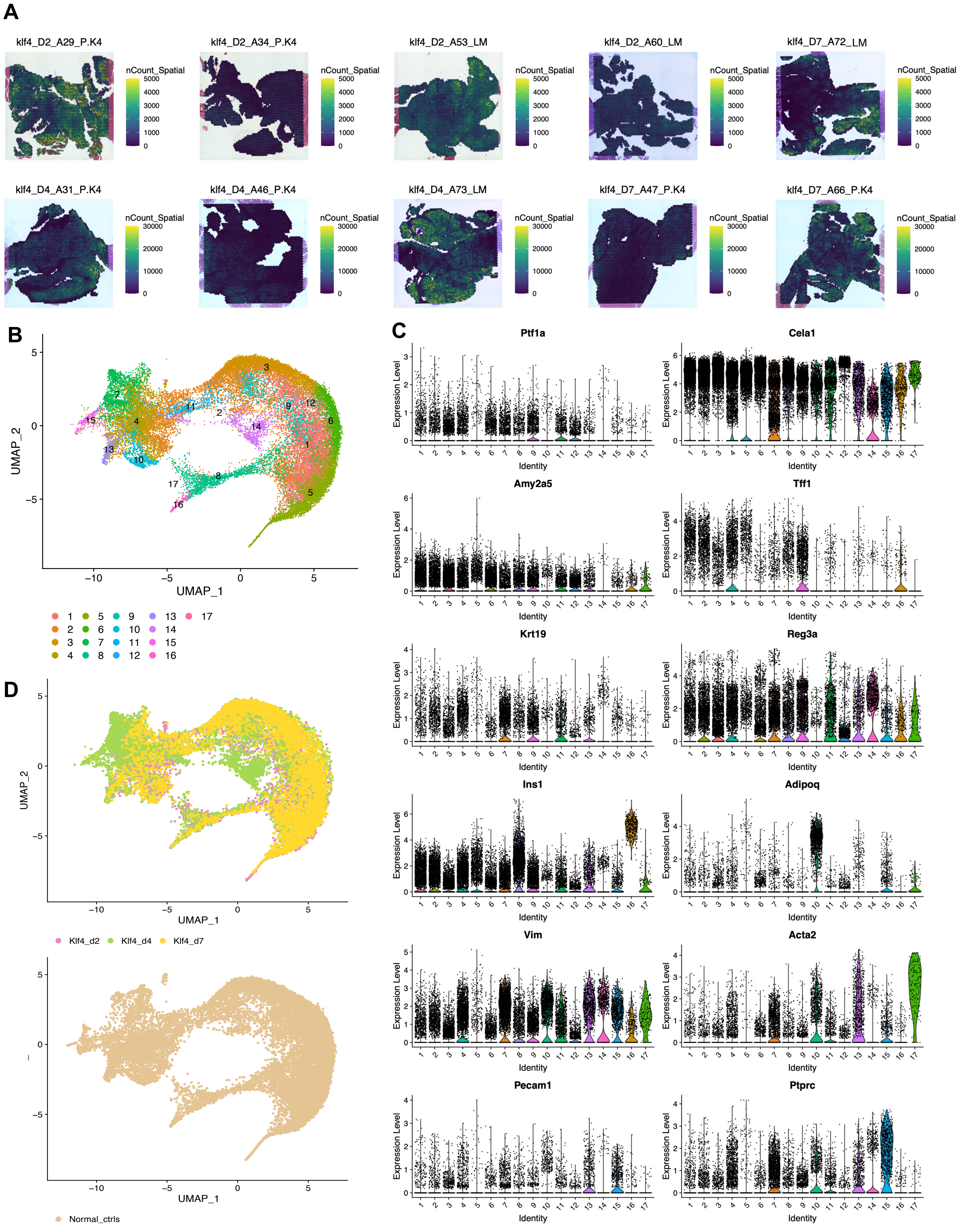

A
klf4_D2_A29_P.K4
klf4_D2_A34_P.K4
klf4_D2_A53_LM
klf4_D2_A60_LM
klf4_D7_A72_LM
nCount_Spatial
klf4_D4_A31_P.K4
klf4_D4_A46_P.K4
klf4_D4_A73_LM
klf4_D7_A47_P.K4
klf4_D7_A66_P.K4
B
UMAP_1
UMAP_2
C
Ptf1a
Cela1
Amy2a5
Tff1
Krt19
Reg3a
Ins1
Adipoq
Vim
Acta2
Pecam1
Ptprc
Expression Level
Identity
D
Klf4_d2
Klf4_d4
Klf4_d7
Normal_ctrls

Figure S3. (Additional File 9). 10X Visium Spatial Transcriptomics profiling.

A. Per-spot count distribution for all AK model pancreata. Normal acinar, n = 4 mice; day 2 ADM, n = 2 mice; day 4 ADM, n = 2 mice; day 7 ADM, n = 2 mice; duct, n = 10 mice.

B. UMAP projection of leiden clustering for all AK model ST spots (n = 31,620 spots).

C. Normalized expression levels of canonical acinar (Ptf1a, Cela1, Amy2a5), duct (Tff1, Krt19), ADM (Reg3a), endocrine (Ins1), adipose (Adipo1), fibroblast (Vim), endothelial (Acta2, Pecam1), and immune (Ptprc) marker genes by cluster.

D. UMAP projection of AK model spots by timepoint (top) and normal control spots (bottom).

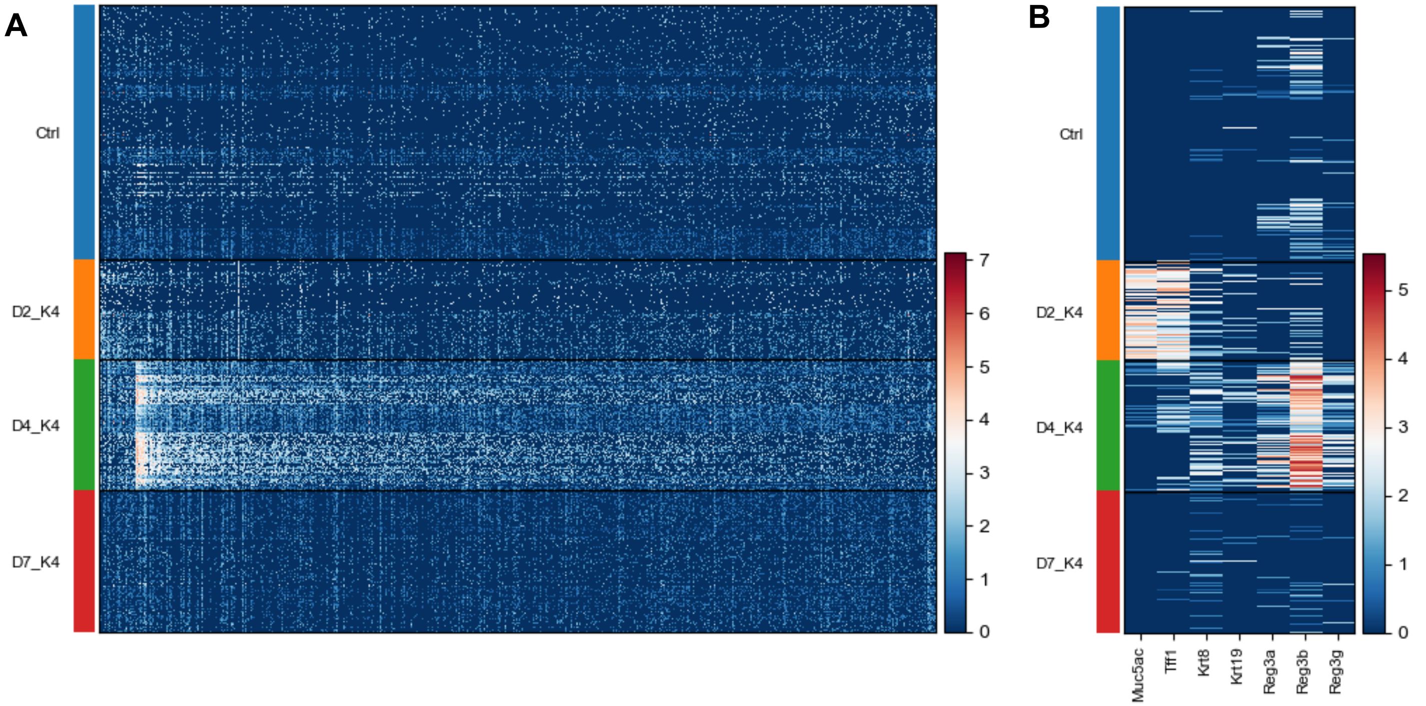

A
Ctrl
D2_K4
D4_K4
D7_K4
7
6
5
4
3
2
1
0
B
Ctrl
D2_K4
D4_K4
D7_K4
5
4
3
2
1
0
Muc5ac
Tff1
Krt8
Krt19
Reg3a
Reg3b
Reg3g

Figure S4. (Additional File 10). Gene expression differences in AK model as profiled by Spatial Transcriptomics.
A. Relative gene expression of genes upregulated at D2, D4, and D7 in AK model versus controls.
B. Relative gene expression of selected marker genes in AK model.

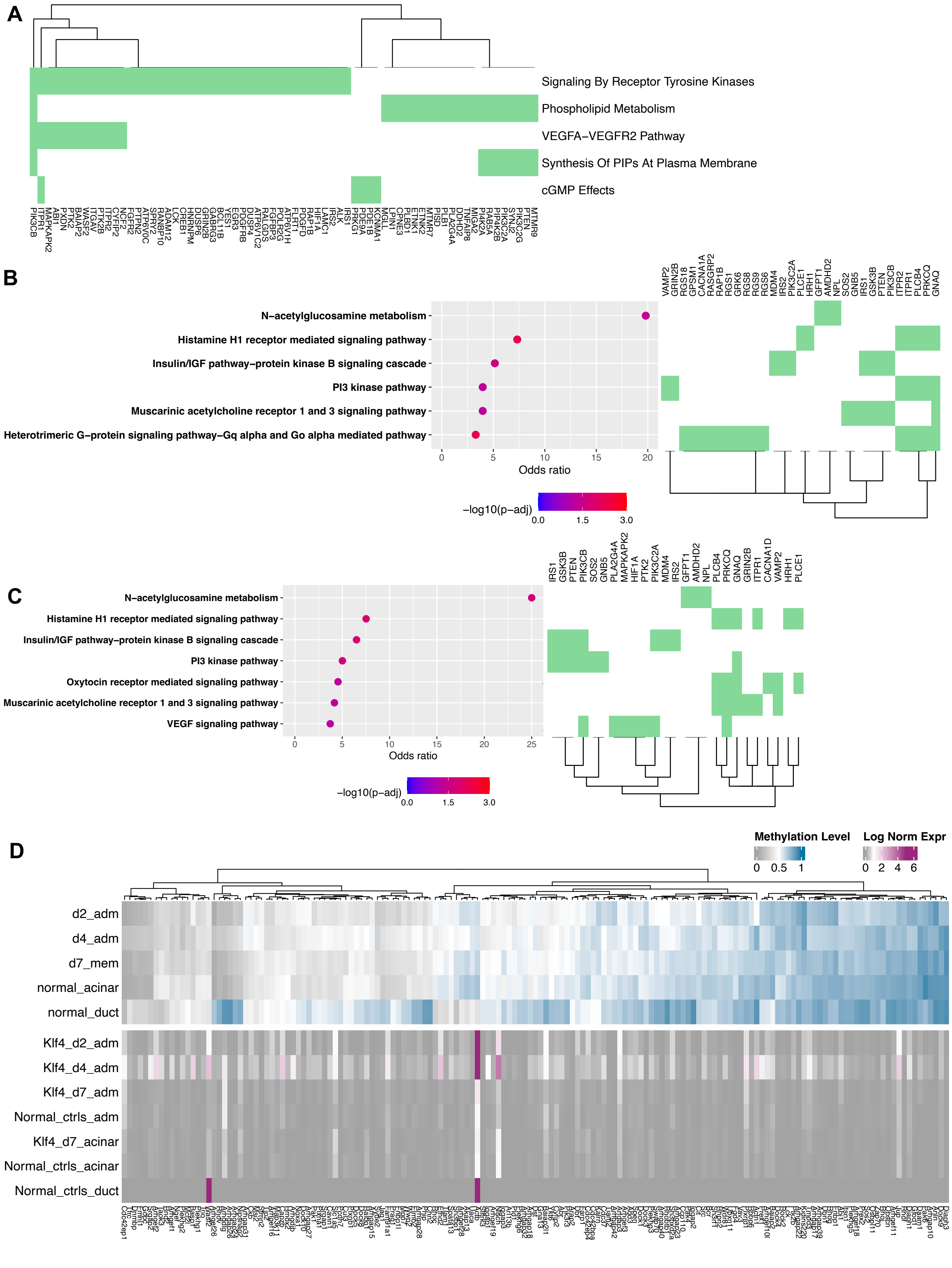
A
Signaling By Receptor Tyrosine Kinases
Phospholipid Metabolism
VEGFA−VEGFR2 Pathway
Synthesis Of PIPs At Plasma Membrane
cGMP Effects
PIK3CB
ITPR1
MAPKAPK2
ABI1
PXDN
PTK2
BAIAP2
WASF2
ITGAV
PTK2B
ITPR2
NCF2
CYFIP2
FGFR2
PTPN2
ATP6V0C
SPRY2
RANBP10
ADAM12
LCK
CREB1
HNRNPM
DUSP6
GABRG3
BCL11B
YES1
EGR3
PDGFRB
DUSP4
ATP6V1C2
RALGDS
FGFBP3
POLR2G
ATP6V1H
FLRT1
RAP1B
HIF1A
LAMC1
IRS2
ALK
IRS1
PRKG1
PDE9A
PDE1B
KCNMA1
MGLL
LPIN1
CPNE3
PLBD1
ETNK1
ETNK2
MTMR7
PISD
PLB1
PLA2G4A
DDHD2
TNFAIP8
MIGA2
PI4K2A
RAB5A
PIP4K2B
PIK3C2A
SYNJ2
PIK3C2G
PTEN
MTMR9
B
N−acetylglucosamine metabolism
Histamine H1 receptor mediated signaling pathway
Insulin/IGF pathway−protein kinase B signaling cascade
PI3 kinase pathway
Muscarinic acetylcholine receptor 1 and 3 signaling pathway
Heterotrimeric G−protein signaling pathway−Gq alpha and Go alpha mediated pathway
0
5
10
15
20
Odds ratio
−log10(p−adj)
0.0
1.5
3.0
VAMP2
GRIN2B
RGS18
GPSM1
CACNA1A
RASGRP2
RAP1B
RGS1
GRK6
RGS8
RGS9
RGS6
MDM4
IRS2
PIK3C2A
PLCE1
HRH1
GFPT1
AMDHD2
NPL
SOS2
GNB5
IRS1
GSK3B
PTEN
PIK3CB
ITPR2
ITPR1
PLCB4
PRKCQ
GNAQ
C
N−acetylglucosamine metabolism
Histamine H1 receptor mediated signaling pathway
Insulin/IGF pathway−protein kinase B signaling cascade
PI3 kinase pathway
Oxytocin receptor mediated signaling pathway
Muscarinic acetylcholine receptor 1 and 3 signaling pathway
VEGF signaling pathway
0
5
10
15
20
25
Odds ratio
−log10(p−adj)
0.0
1.5
3.0
IRS1
GSK3B
PTEN
PIK3CB
SOS2
GNB5
PLA2G4A
MAPKAPK2
HIF1A
PTK2
PIK3C2A
MDM4
IRS2
GFPT1
AMDHD2
NPL
PLCB4
PRKCQ
GNAQ
GRIN2B
ITPR1
CACNA1D
VAMP2
HRH1
PLCE1
D
Methylation Level
0
0.5
1
Log Norm Expr
0
2
4
6
d2_adm
d4_adm
d7_mem
normal_acinar
normal_duct
Klf4_d2_adm
Klf4_d4_adm
Klf4_d7_adm
Normal_ctrls_adm
Klf4_d7_acinar
Normal_ctrls_acinar
Normal_ctrls_duct

Figure S5. (Additional File 12). Memory DMR profiles for AK model pancreata.

A. Genes (columns) overlapping AK model D7 vs normal acinar DMRs that contribute to each enriched Reactome gene set (rows) as in Fig. 5B.

B. Fisher's GSEA of genes overlapping AK model D7 vs normal acinar DMRs using Panther 2016 gene sets (left), with corresponding genes contributing to each gene set (right).

C. Fisher's GSEA of genes overlapping AK model D7 vs normal acinar DMRs for which the average D7 methylation value was more extreme than that of D2 & D4 ADM relative to normal acini controls. Fisher's GSEA performed with Panther 2016 gene sets (left), with corresponding genes contributing to each gene set (right).

D. Summary heatmap of methylation level and normalized expression level at Reactome- and KEGG-annotated Rho/Rac/Cdc42 GTPase-related genes for each timepoint. Normal acinar, n = 4 mice; day 2 ADM, n = 2 mice; day 4 ADM, n = 2 mice; day 7 ADM, n = 2 mice; duct, n = 10 mice.

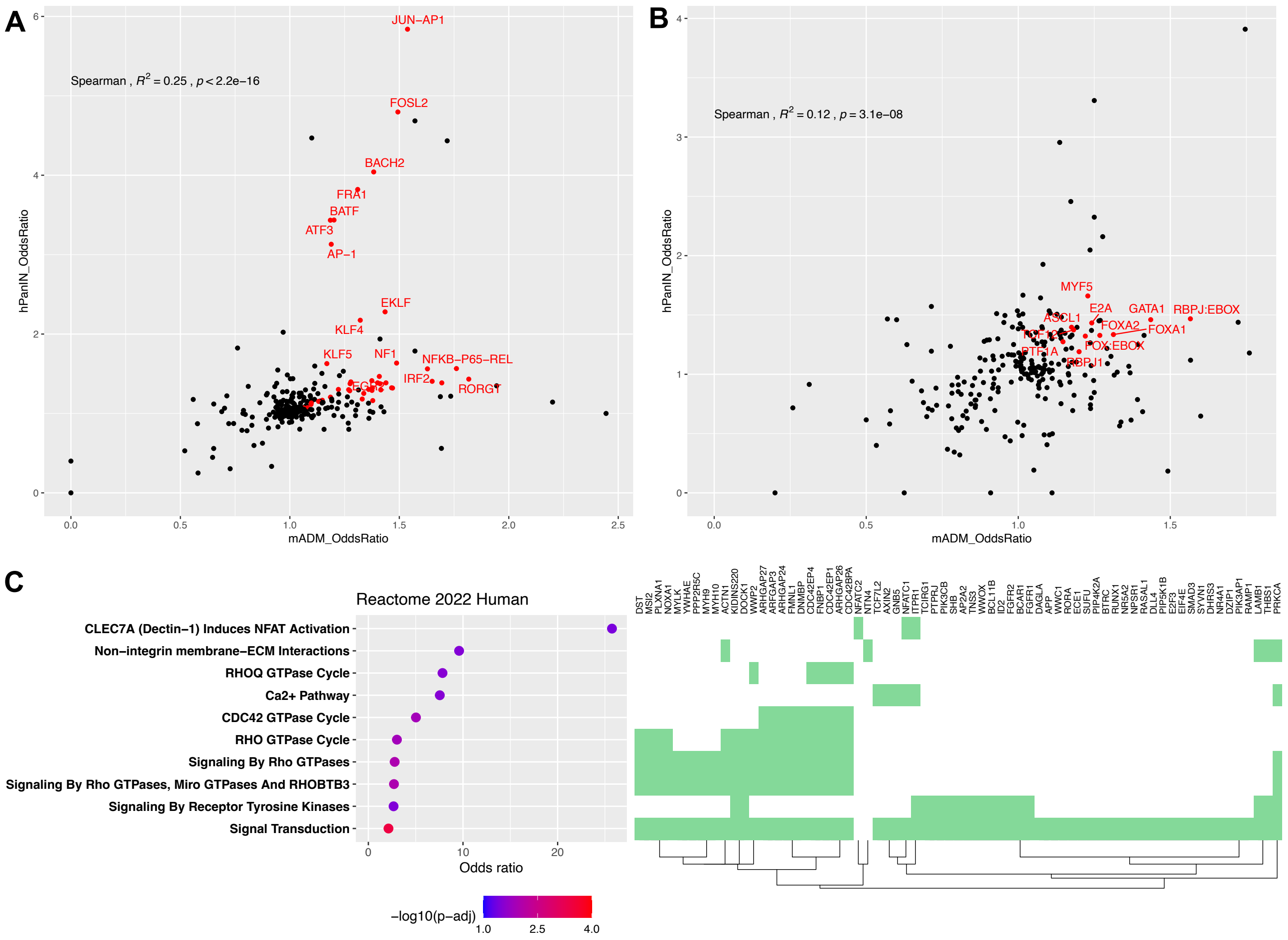
A
Spearman , $R^2 = 0.25$, $p < 2.2e{-}16$
JUN–AP1
FOSL2
BACH2
FRA1
BATF
ATF3
AP–1
EKLF
KLF4
KLF5
NF1
NFKB–P65–REL
IRF2
RORGT
hPanIN_OddsRatio
mADM_OddsRatio
B
Spearman , $R^2 = 0.12$, $p = 3.1e{-}08$
MYF5
E2A
GATA1
RBPJ:EBOX
ASCL1
FOXA2
FOXA1
FOX:EBOX
PTF1A
RBPJ1
hPanIN_OddsRatio
mADM_OddsRatio
C
Reactome 2022 Human
CLEC7A (Dectin–1) Induces NFAT Activation
Non–integrin membrane–ECM Interactions
RHOQ GTPase Cycle
Ca2+ Pathway
CDC42 GTPase Cycle
RHO GTPase Cycle
Signaling By Rho GTPases
Signaling By Rho GTPases, Miro GTPases And RHOBTB3
Signaling By Receptor Tyrosine Kinases
Signal Transduction
Odds ratio
–log10(p–adj)
DST MSI2 PLXNA1 NOXA1 MYLK YWHAE PPP2R5C MYH9 MYH10 ACTN1 KIDINS220 DOCK1 WWP2 ARHGAP27 ARFGAP3 ARHGAP24 FMNL1 DNMBP CDC42EP4 FNBP1 CDC42EP1 ARHGAP26 CDC42BPA NFATC2 NTN4 TCF7L2 AXIN2 GNB5 NFATC1 ITPR1 TCIRG1 PTPRJ PIK3CB SHB AP2A2 TNS3 WWOX BCL11B ID2 FGFR2 BCAR1 FGFR1 DAGLA APP WWC1 RORA ECE1 SUFU PIP4K2A BTRC RUNX1 NR5A2 NPSR1 RASAL1 DLL4 PIP5K1B E2F3 EIF4E SMAD3 SYVN1 DHRS3 NR4A1 DZIP1 PIK3AP1 RAMP1 LAMB1 THBS1 PRKCA

Figure S6. (Additional File 14). Consistent patterns among ADM DMRs and human PanIN DMRs.

A-B. Correlation of motif enrichment analysis between mouse ADM and human PanIN.

A. X-axis: motif enrichment odds ratio for mouse AK model D4 ADM vs acinar *hypomethylated* DMRs. Y-axis: motif enrichment odds ratio for human PanIN vs acinar *hypomethylated* DMRs.
B. X-axis: motif enrichment odds ratio for mouse AK model D4 ADM vs acinar *hypermethylated* DMRs. Y-axis: motif enrichment odds ratio for human PanIN vs acinar *hypermethylated* DMRs.
C. GSEA of shared DMR genes between mouse AK model D4 ADM vs acinar and human PanIN vs acinar (left), with genes contributing to each enriched gene set (right).

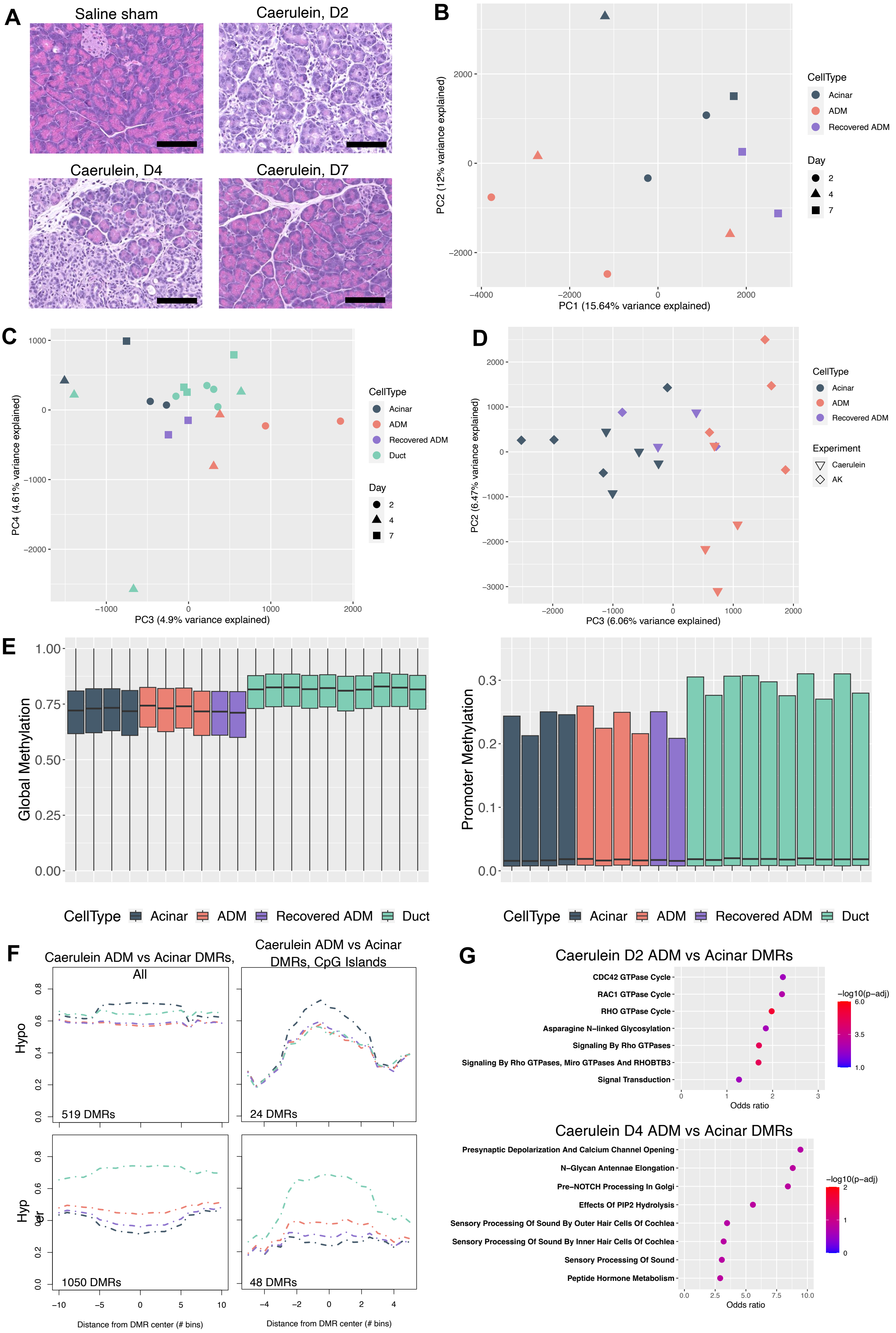
A
Saline sham
Caerulein, D2
Caerulein, D4
Caerulein, D7
B
PC1 (15.64% variance explained)
PC2 (12% variance explained)
CellType
Acinar
ADM
Recovered ADM
Day
2
4
7
C
PC3 (4.9% variance explained)
PC4 (4.61% variance explained)
CellType
Acinar
ADM
Recovered ADM
Duct
Day
2
4
7
D
PC3 (6.06% variance explained)
PC2 (6.47% variance explained)
CellType
Acinar
ADM
Recovered ADM
Experiment
Caerulein
AK
E
Global Methylation
Promoter Methylation
CellType Acinar ADM Recovered ADM Duct
CellType Acinar ADM Recovered ADM Duct
F
Caerulein ADM vs Acinar DMRs, All
Caerulein ADM vs Acinar DMRs, CpG Islands
Hypo
519 DMRs
24 DMRs
1050 DMRs
48 DMRs
Distance from DMR center (# bins)
Distance from DMR center (# bins)
G
Caerulein D2 ADM vs Acinar DMRs
CDC42 GTPase Cycle
RAC1 GTPase Cycle
RHO GTPase Cycle
Asparagine N-linked Glycosylation
Signaling By Rho GTPases
Signaling By Rho GTPases, Miro GTPases And RHOBTB3
Signal Transduction
Odds ratio
−log10(p−adj)
Caerulein D4 ADM vs Acinar DMRs
Presynaptic Depolarization And Calcium Channel Opening
N-Glycan Antennae Elongation
Pre-NOTCH Processing In Golgi
Effects Of PIP2 Hydrolysis
Sensory Processing Of Sound By Outer Hair Cells Of Cochlea
Sensory Processing Of Sound By Inner Hair Cells Of Cochlea
Sensory Processing Of Sound
Peptide Hormone Metabolism
Odds ratio
−log10(p−adj)

Figure S7. (Additional File 15). Methylation profiles in widely used caerulein model of pancreatitis.

A. Hematoxylin and eosin (H&E) staining of normal control, and Day 2, Day 4, and Day 7 pancreata in caerulein model of acute pancreatitis.
B. PCA of laser capture microdissection-purified acini, ADM lesions, and recovered ADM lesions at PCs 1 and 2.
C. PCA of laser capture microdissection-purified acini, ADM lesions, recovered ADM lesions, and ducts at PCs 3 and 4.
D. PCA of laser capture microdissection-purified acini, ADM lesions, recovered ADM lesions combined from both the AK and caerulein experiments at PCs 2 and 3.
E. Boxplot of genome-wide (left) and promoter-specific (right) CpG methylation values for all exocrine samples.
F. Meta-region plots of caerulein model ADM vs acinar DMRs summarized across all regions (left) and across CpG islands only (right) with 50% width buffer on each side.
G. GSEA of genes overlapping caerulein model D2 ADM vs acinar DMRs (top) and D4 ADM vs acinar DMRs (bottom) using Reactome 2022 Human gene sets.

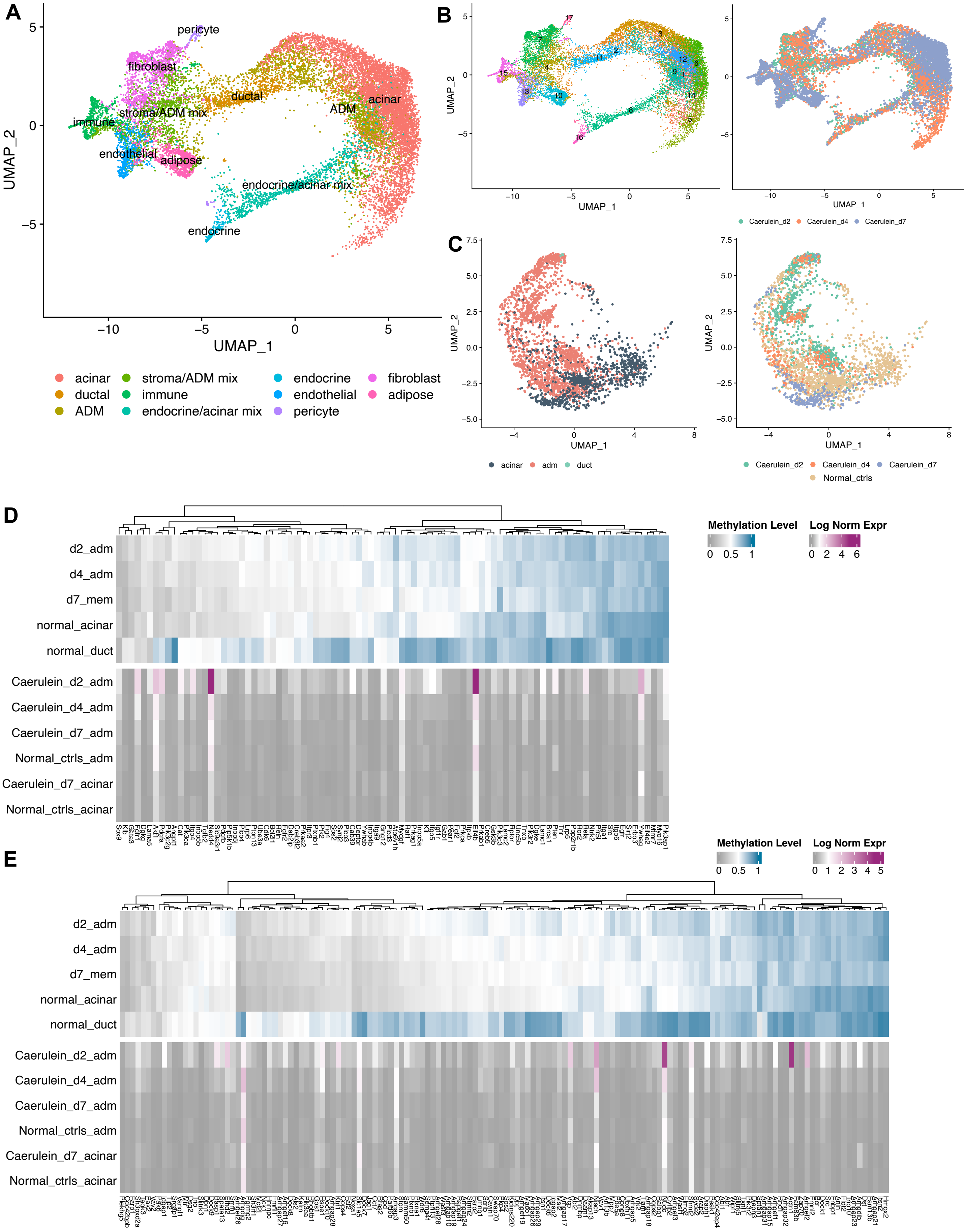

A
pericyte
fibroblast
stroma/ADM mix
immune
ductal
ADM
acinar
endothelial
adipose
endocrine/acinar mix
endocrine
UMAP_1
UMAP_2
acinar
ductal
ADM
stroma/ADM mix
immune
endocrine/acinar mix
endocrine
endothelial
pericyte
fibroblast
adipose
B
UMAP_1
UMAP_2
Caerulein_d2
Caerulein_d4
Caerulein_d7
C
UMAP_1
UMAP_2
acinar
adm
duct
Caerulein_d2
Caerulein_d4
Caerulein_d7
Normal_ctrls
D
Methylation Level
Log Norm Expr
d2_adm
d4_adm
d7_mem
normal_acinar
normal_duct
Caerulein_d2_adm
Caerulein_d4_adm
Caerulein_d7_adm
Normal_ctrls_adm
Caerulein_d7_acinar
Normal_ctrls_acinar
E
Methylation Level
Log Norm Expr
d2_adm
d4_adm
d7_mem
normal_acinar
normal_duct
Caerulein_d2_adm
Caerulein_d4_adm
Caerulein_d7_adm
Normal_ctrls_adm
Caerulein_d7_acinar
Normal_ctrls_acinar

Figure S8. (Additional File 16). Gene expression patterns in caerulein model pancreata.

A. UMAP projection of caerulein model Visium ST spots (n = 16,079 spots) by manually annotated major cell type.
B. UMAP projection of ST spots by leiden clustering (left) and timepoint (right) for all caerulein model samples.
C. UMAP projection of high-purity exocrine spots from caerulein model pancreata by deconvolution-assigned cell type (left) and by timepoint (right).
D. Summary heatmap of methylation level and normalized expression level at Reactome- and KEGG-annotated Pi3k GTPase-related genes for each caerulein sample.
E. Summary heatmap of methylation level and normalized expression level at Reactome- and KEGG-annotated Rho/Rac/Cdc42 GTPase-related genes for each caerulein sample.

Normal acinar, n = 4 mice; day 2 ADM, n = 2 mice; day 4 ADM, n = 2 mice; day 7 ADM, n = 2 mice; duct, n = 10 mice.

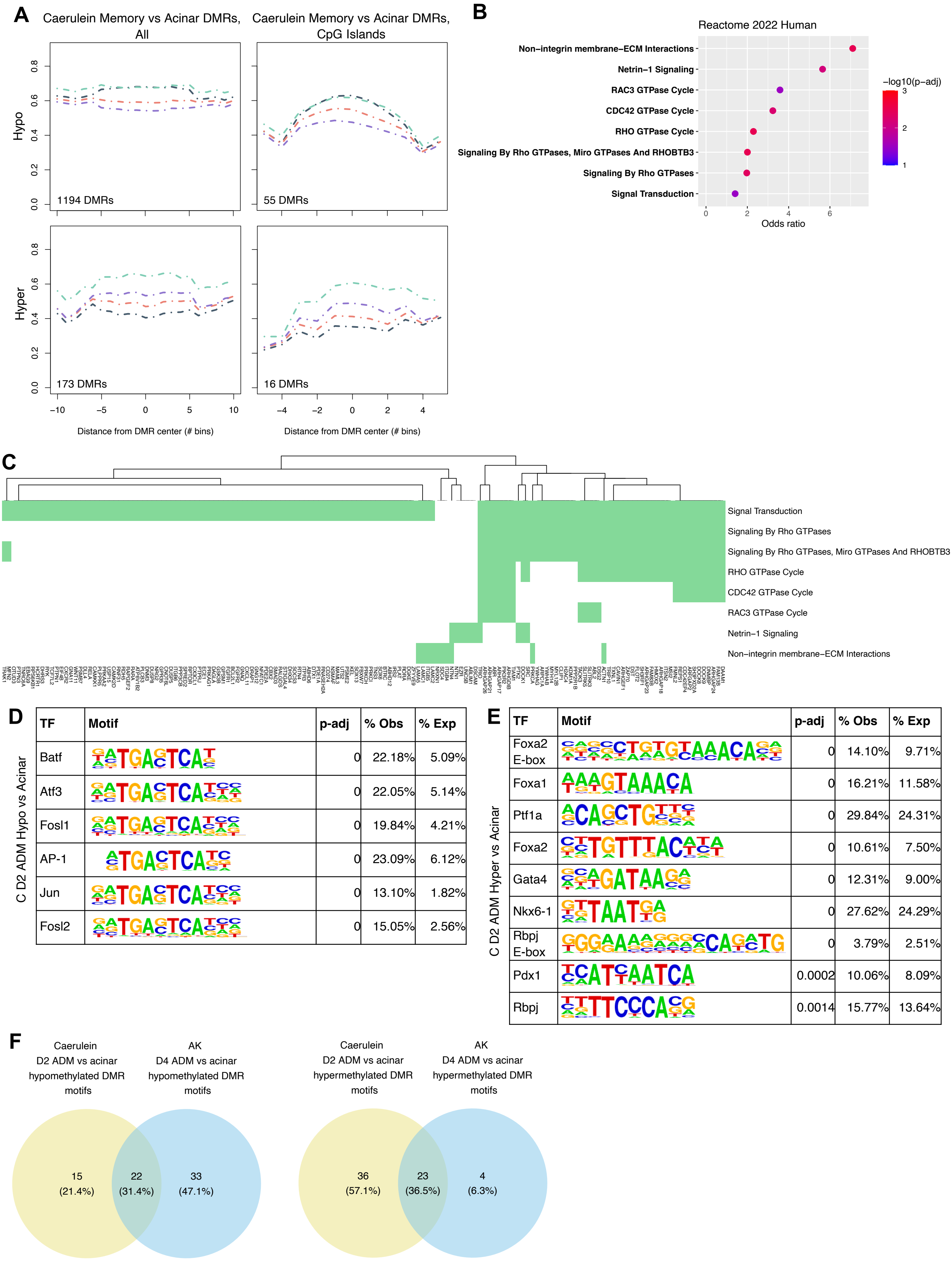

A
Caerulein Memory vs Acinar DMRs, All
Caerulein Memory vs Acinar DMRs, CpG Islands
Hypo
Hyper
1194 DMRs
55 DMRs
173 DMRs
16 DMRs
Distance from DMR center (# bins)
B
Reactome 2022 Human
Non−integrin membrane−ECM Interactions
Netrin−1 Signaling
RAC3 GTPase Cycle
CDC42 GTPase Cycle
RHO GTPase Cycle
Signaling By Rho GTPases, Miro GTPases And RHOBTB3
Signaling By Rho GTPases
Signal Transduction
Odds ratio
−log10(p−adj)
C
Signal Transduction
Signaling By Rho GTPases
Signaling By Rho GTPases, Miro GTPases And RHOBTB3
RHO GTPase Cycle
CDC42 GTPase Cycle
RAC3 GTPase Cycle
Netrin−1 Signaling
Non−integrin membrane−ECM Interactions
D
C D2 ADM Hypo vs Acinar
TF | Motif | p-adj | % Obs | % Exp
Batf | | 0 | 22.18% | 5.09%
Atf3 | | 0 | 22.05% | 5.14%
Fosl1 | | 0 | 19.84% | 4.21%
AP-1 | | 0 | 23.09% | 6.12%
Jun | | 0 | 13.10% | 1.82%
Fosl2 | | 0 | 15.05% | 2.56%
E
C D2 ADM Hyper vs Acinar
TF | Motif | p-adj | % Obs | % Exp
Foxa2 E-box | | 0 | 14.10% | 9.71%
Foxa1 | | 0 | 16.21% | 11.58%
Ptf1a | | 0 | 29.84% | 24.31%
Foxa2 | | 0 | 10.61% | 7.50%
Gata4 | | 0 | 12.31% | 9.00%
Nkx6-1 | | 0 | 27.62% | 24.29%
Rbpj E-box | | 0 | 3.79% | 2.51%
Pdx1 | | 0.0002 | 10.06% | 8.09%
Rbpj | | 0.0014 | 15.77% | 13.64%
F
Caerulein D2 ADM vs acinar hypomethylated DMR motifs
AK D4 ADM vs acinar hypomethylated DMR motifs
15 (21.4%)
22 (31.4%)
33 (47.1%)
Caerulein D2 ADM vs acinar hypermethylated DMR motifs
AK D4 ADM vs acinar hypermethylated DMR motifs
36 (57.1%)
23 (36.5%)
4 (6.3%)

Figure S9. (Additional File 17). DNA methylation memory in caerulein model of ADM.

A. Meta-region plots of caerulein model D7 vs acinar DMRs summarized across all regions (left) and across CpG islands only (right) with 50% width buffer on each side. Normal acinar, n = 4 mice; day 2 ADM, n = 2 mice; day 4 ADM, n = 2 mice; day 7 ADM, n = 2 mice; duct, n = 10 mice.
B. GSEA of genes overlapping caerulein model D7 vs acinar DMRs using Reactome 2022 Human gene sets.
C. Genes (columns) overlapping caerulein model D7 ADM vs normal acinar DMRs that contribute to each significantly enriched gene set (rows) as in B.
D. Motif enrichment analysis results at caerulein ADM vs acinar hypo DMRs at Day 2.
E. Motif enrichment analysis results at caerulein ADM vs acinar hyper DMRs at Day 2.
F. Venn diagrams indicating the overlap of hypomethylated DMR motifs and hypermethylated DMR motifs between the Caerulein and AK models.

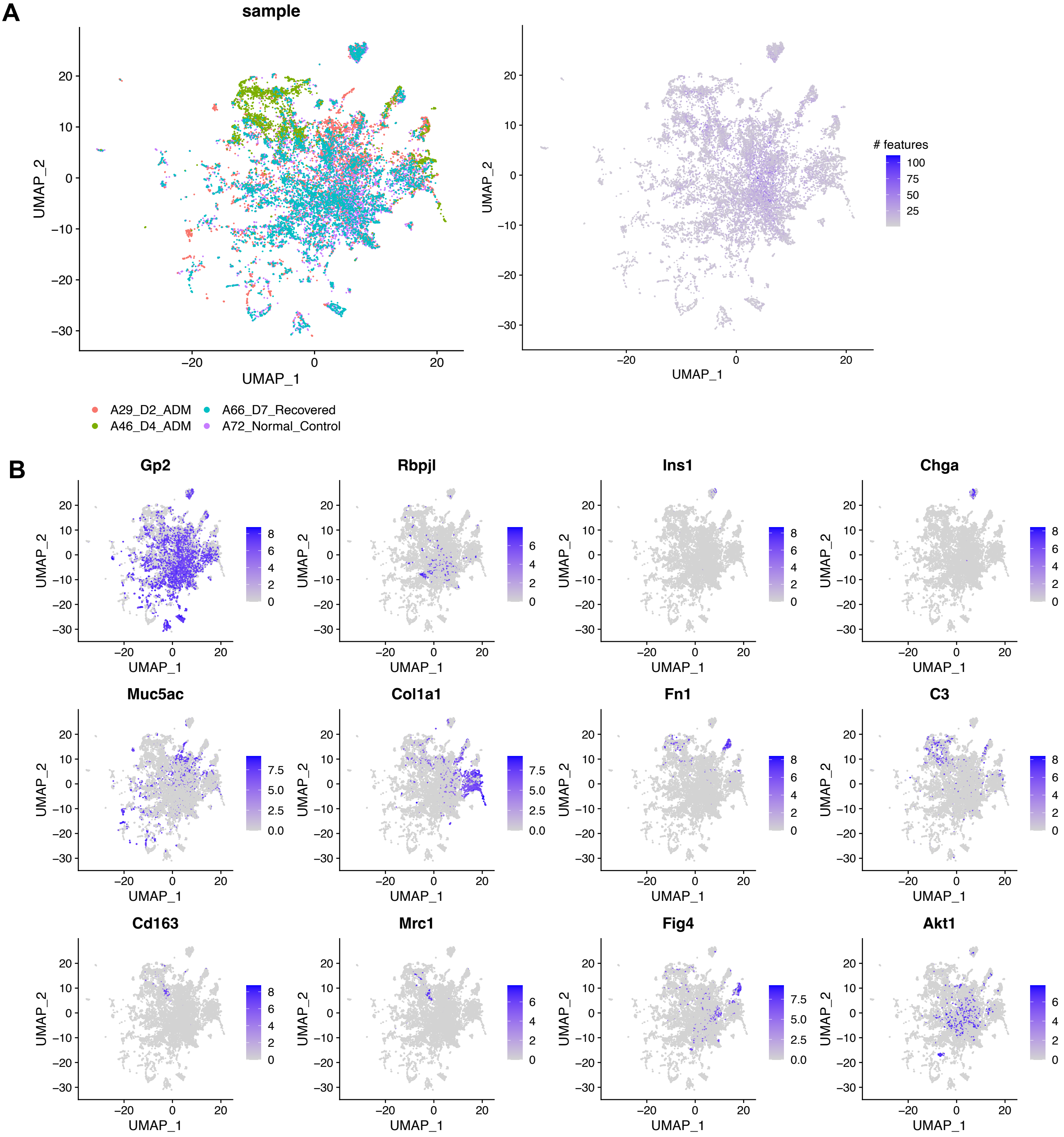
A
sample
UMAP_1
UMAP_2
features
A29_D2_ADM
A46_D4_ADM
A66_D7_Recovered
A72_Normal_Control
B
Gp2
Rbpjl
Ins1
Chga
Muc5ac
Col1a1
Fn1
C3
Cd163
Mrc1
Fig4
Akt1

Figure S10. (Additional File 18). MERSCOPE targeted spatial transcriptomics on AK model pancreata.

A. UMAP projection of all MERSCOPE cell profiles (n = 19776 cells) by timepoint (left) and by number of genes detected in each cell (right)

B. Normalized expression of canonical marker genes for acini (*Gp2, Rbpjl*), islet cells (*Ins1, Chga*), PanINs (*Muc5ac*), fibroblasts (*Col1a1, Fn1*), macrophages (*Cd163, Mrc1*), and the Pi3k pathway (*Fig4, Akt1*).
